# Comparative Study on the Hypoglycemic Effects of Different Parts of *Musa balbisiana*

**DOI:** 10.1002/fsn3.4573

**Published:** 2024-11-07

**Authors:** Thi Ngoc Nhon Hoang, Quang Liem Nguyen, Thi Thanh Ngan Le, Ngoc Hoa Vo, Thi Anh Dao Dong, Thi Hong Anh Le

**Affiliations:** ^1^ Faculty of Food Science and Technology Ho chi Minh City University of Industry and Trade (HUIT) Ho Chi Minh City Vietnam; ^2^ Department of Food Technology, Faculty of Chemical Engineering Ho chi Minh City University of Technology (HCMUT) Ho Chi Minh City Vietnam; ^3^ Vietnam National University Ho Chi Minh City Ho Chi Minh City Vietnam

**Keywords:** blood glucose index, hypoglycemia, *Musa balbisiana*, α‐amylase, α‐glucosidase

## Abstract

Diabetes mellitus is a chronic metabolic disorder that can cause elevated blood glucose levels due to impaired insulin secretion or resistance. Different parts of *Musa balbisiana* have been used widely in traditional medicine to treat many disorders. The present study aims to evaluate the antidiabetic ability of the corm, pseudostem, inflorescence, fruit, peel, and seed of *M. balbisiana* via in vitro experiments by inhibiting α‐amylase and α‐glucosidase enzymes as well as in vivo models on diabetic alloxan‐induced mice. The results show that all investigated parts have performed potential inhibition on two investigated digestive enzymes. Seed poses the highest capacity among surveyed parts on α‐amylase (IC_50_:f μg/mL) and α‐glucosidase (IC_50_: 21.63 μg/mL) as well as effectively lowers the blood glucose index (IG) in alloxan‐induced mice. In addition, fruit, corm, and inflorescence are considered essential parts that have high hypoglycemic effects via in vivo experiments. These findings indicate that all *M. balbisiana* parts are possibly a potential source for hypoglycemic agents; further clinical studies are needed to evaluate the safety of human beings before applying them in functional food and pharmaceutical industries.

## Graphical abstract

The antidiabetic ability in vitro and in vivo of the corm, pseudostem, inflorescence, fruit, peel, and seed of *M. balbisiana* was determined. All investigated parts have performed potential antidiabetic activity on inhibition enzymes of α‐amylase and α‐glucosidase. Fruit, corm, inflorescence, and seed samples are considered essential parts that have high hypoglycemic effects, as shown by in vivo experiments. Seed poses the highest capacity among surveyed parts on inhibition of two investigated digestive enzymes as well as effectively lowering the blood glucose level in alloxan‐induced mice. The corm, pseudostem, inflorescence, fruit, peel, and seed of *M. balbisiana* performed potential antidiabetic activity on inhibition enzymes of α‐amylase and α‐glucosidase.

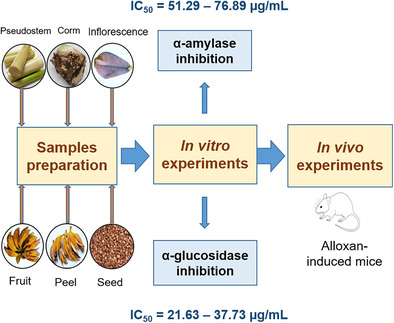

## 1 Introduction

Diabetes is considered one of the biggest health catastrophes in the world, which causes enormous burdens on patients and communities. As reported by the IDF (International Diabetes Federation) Diabetes Atlas, the world's diabetic patients number was 537 million in 2021, and this figure is projected to reach 643 million by 2030 and 783 million by 2045 (Sun et al. 2022). Thus, the increase in the number of diabetes mellitus will enlarge the national and international healthcare budget. Over 1.2 million children and adolescents had Type 1 diabetes in 2021. The direct health expenditure due to diabetes is already close to one trillion USD, and this cost will be much higher by 2030 (Magliano and Boyko 2022). Vietnam is in the top 10 countries with the highest increase rate of diabetes cases. According to the Vietnamese Association of Diabetes and Endocrinology (VADE), about 6% of Vietnamese people have been living with diabetes. And the number is estimated to be 7 or 8 million by 2025 (Duc et al. 2023). This phenomenon will bring remarkable trouble to the national healthcare budget. Currently, some strategies are being applied for diabetes mellitus treatment, such as diet, exercise, various oral antidiabetic drugs, and insulin therapy.

Diabetes mellitus, a metabolic disorder disease, relates to high blood sugar levels for a prolonged period and disturbances in carbohydrate, lipid, and protein metabolism. It results from abnormalities of insulin secretion, insulin work, or both (Bhaskar et al. 2011). Two key enzymes, α‐amylase and intestinal α‐glucosidases, play a vital role in carbohydrate digestion and absorption. The lack of glucagon, which catalyzes the accumulation of sugar into glycogen in the liver and muscles, as well as the lack of insulin to bring sugar to cells to produce energy, causes hyperglycemia in diabetic patients (Ramu et al. 2015). During diabetes, hyperglycemia causes the generation of free radicals, leading to oxidative stress. This condition might cause a few dangerous microvascular and macrovascular complications, namely ephropathy, neuropathy, retinopathy, and cardiovascular diseases (Bhaskar et al. 2011). Diabetic patients have to use drugs to maintain the glucose in their blood. However, synthetic hypoglycemic agents can bring several adverse effects, which include hypoglycemia, frequent diarrhea, hypertension, hypercoagulability, lactic acidosis, hepatotoxicity, and dyslipidemia. A variety of phytochemicals are present in herbal medicines, which have been used for traditional treatment of a wide range of diseases. It is believed that they are naturally safe and effective, with fewer side effects (Ansari et al. 2022). There is a growing interest in more effective and safer hypoglycemic agents that might also be for babies or women in pregnancy. Thus, these researchers pay much attention to novel therapeutic agents, preferably from natural sources. Plant‐based antidiabetic agents have been extensively developed for phytopharmaca (Shanmuga and Subramanian 2012). Phytomedicine can help diabetic patients with fewer side effects and lower expenditure on treatment. The natural remedies are less harmful to the body than synthetic ones. Some plants have a strong antidiabetic effect and are well known in the community (Ara, Tripathy, and Ghosh 2019). In addition, using medicinal plants in diabetes mellitus management could be a good solution for people with low‐cost income or rural, especially in remote areas of developing countries with poorly served health facilities. Among numerous traditional medicinal plants reported to have hypoglycemic properties, *Musa balbisiana* is one of the most common materials used in Vietnamese traditional medicine. The local people use juice from the aerial stem and rhizome of *M. balbisiana* as antidiabetic medicines. *M. balbisiana* is originally from wild areas and is commonly known as “banana seeds” in Vietnam. An alkaline solution was created using ash powder obtained from various parts, such as pseudostem, corm, peel, and other parts, and it was also used in many dishes. Daily consumption of a slice of ripe fruit that has been soaked in water or wine and infused with water or wine is beneficial for good health. Dysentery and watery defecation can be cured by consuming a slice of unripe fruit mixed with salt. The inflorescence extract can be consumed on an empty stomach to treat stomach aches and helminth infections. Boiling the inflorescence's extract is also used for treating jaundice (Swargiary et al. 2021).

Different parts of *Musa* spp. have been used in traditional folklore medicine for various medicinal purposes, including the treatment of diabetes mellitus in several ways, such as diet therapy, which can work different mechanisms like slowing down sugar absorption in the gastro‐intestinal tract, increasing insulin production in β cells in the pancreas, or by mimicking insulin activity. Banana flowers and pseudostem are rich in bioactive compounds (Bhaskar et al. 2011). The juice of the *Musa sapientum* flowers or its extract was determined to have beneficial effects in reducing blood glucose, glycosylated hemoglobin, and oral glucose tolerance in alloxan‐induced diabetic rats, which is possibly related to antioxidant activity to prove the folklore claim scientifically (Eleazu, Iroaganachi, and Eleazu 2013), (Dhanabal et al. 2005). Low amounts of minerals and sugars are present in unripe *Musa paradisiaca*, but it is being scientifically documented as a hypoglycemic plant (Eleazu and Okafor 2012). *Musa* spp. fruits are a source of several bioactive components and are considered one of the attractive targets for antidiabetic studies. *Musa* pseudostem, with its antioxidant activity, reveals remarkable inhibition of mammalian intestinal α‐glucosidases and yeast α‐glucosidase (IC_50_, 8.11 ± 0.10 μg/mL). Its extract inhibited sucrase, maltase, and p‐nitrophenyl‐α‐D‐glucopyranoside hydrolysis by mixed‐type inhibition (Ramu et al. 2015).

*Musa balbisiana* Colla has high medicinal properties and is commonly grown in Vietnam. Its flowers, fruits, stem, roots, and leaves have been used in traditional medicine for many purposes, such as diabetes, cardiovascular disease, and inflammation. Among the health benefits, it is well known for the treatment of diabetes mellitus due to its effects in reducing blood sugar, and this is claimed by local practitioners (Swargiary et al. 2021). However, in the absence of systemic reports in the literature, the present study aimed to evaluate the antidiabetic properties of parts of *M. balbisiana* in vitro and in vivo. This is the first report on the inhibition of all parts such as corm, pseudostem, inflorescence, fruit, peel, and the seed of *M. balbisiana* on enzyme α‐amylase and α‐glucosidase and the effects on alloxan‐induced mice. The study offers the primary platform and worthy information for more clinical studies on the further applications of *M. balbisiana* parts in pharmaceutical products in terms of diabetic problems.

## 2 Materials and Methods

### 2.1 Materials

Parts including corm, pseudostem, inflorescence, fruit, peel, and seed were collected from *M. balbisiana* at An Hoa ward, Tam Nong district, Dong Thap province, Vietnam (the coordinates 10°44′38″ N 105°23′7″ E). Fruits about 80%–85% in maturity were harvested from the tree after 115–120 days of blossom. Then, seed and peel were taken from these fruits. Both corm and pseudostem were peaked up in the same tree after harvesting fruits. The inflorescence was collected from the tree in full bloom, which was the male flower 15 cm away from the bunch of bananas. These parts were rinsed with tap water, followed by distilled water to remove the dirt on the surface. The pseudostem was kept fresh while some parts of the corm, inflorescence, peel, and seeds were sliced and dried at 60°C until below 10% moisture, ground to powder (20–40 mesh size), and kept in zipper bags for sample preparation all experiments.

Dinitrosalicylic acid (Sigma–Aldrich), Acarbose (Sigma–Aldrich), Starch (soluble, Sigma–Aldrich), p‐nitrophenyl‐α‐D‐glucopyranoside (Sigma–Aldrich), α‐glucosidase enzyme (Sigma–Aldrich), α‐amylase enzyme (Sigma–Aldrich). Methanol (Merck), α‐glucosidase enzyme (Merck), H_2_SO_4_ (Merck), chloroform (Merck), dimethyl sulfoxide (DMSO, Merck). Other chemicals and reagents were of analytical grade.

### 2.2 Samples Preparation

To determine the diabetic properties of parts of *M. balbisiana*, these parts, including corm, inflorescence, fruit, peel, and seed, were extracted according to the protocol in our previous study (Nhon Hoang et al. 2023). Briefly, powder materials were added with 70% methanol, with the material/solvent ratios 1/20 w/v, and put in a water bath at 60°C for 120 min. After that, the obtained mixtures were centrifuged at 5500 rpm for 15 min and then filtered through the Whatman No. 4 filter paper to have homogenous extracts. Fresh pseudostem was pressed to obtain pseudostem juice. The obtained powder of corm, pseudostem, inflorescence, fruit, peel, and seed was diluted by dimethyl sulfoxide (DMSO) to have samples in different concentrations for the in vitro experiments and will diluted in distilled water for in vivo experiments.

### 2.3 In Vitro Diabetic Inhibitory Activity of Samples

#### 2.3.1 α‐Amylase Inhibitory Assay

The inhibition of enzyme α‐amylase was carried out according to our previous report (Nhon Hoang et al. 2023). Samples of parts of *M. balbisiana*, including corm, pseudostem, inflorescence, fruit, peel, and seed with different concentrations (20, 40, 60, 80, and 100 μg/mL), were prepared in DMSO 10% from freeze‐drying powder. A mixture of samples (500 μL) and sodium phosphate buffer (pH 6.9, 500 μL of 0.02 M) with sodium chloride 0.006 M containing α‐amylase (20 U/mL) solution was incubated at 37°C for 10 min. Then, add a starch solution (1%, 500 μL) dissolved in 0.02 M sodium phosphate buffer (pH 6.9 with 0.006 M NaCl) to each test tube and continue keeping it at 37°C for 20 min. The reaction mixture was added to 1 mL of a color reagent of dinitrosalicylic acid. The test tubes were then put in a boiling water bath for 5 min to stop the reaction and cooled to room temperature. The reaction mixture was then diluted with 10 mL of distilled water, and absorbance was read at 540 nm. A similar protocol was performed for the positive control—acarbose. The inhibitory activity IC_50_ was expressed as the half‐maximal inhibitory concentration. The α‐amylase inhibition (%) is calculated via the following equation.

$$\%\text{inhibition}=\frac{{\mathrm{Abs}}_{\text{control}}-{\mathrm{Abs}}_{\text{sample}}}{{\mathrm{Abs}}_{\text{control}}}\times 100\%$$

#### 2.3.2 α‐Glucosidase Inhibition Assay

The inhibition of enzyme α‐glucosidase was conducted with the description in our previous report (Nhon Hoang et al. 2023). Six samples with different concentrations (10, 20, 30, 40, and 50 μg/mL) were prepared in DMSO 10% from freeze‐drying powder of different parts of *M. balbisiana* (corm, pseudostem, inflorescence, fruit, peel, seed). A mixture of samples (50 μL) and the control—acarbose and 0.1 M phosphate buffer (100 μL, pH 6.9) containing α‐glucosidase solution (1 U/mL) was incubated in 96‐well plates at 25°C for 10 min. After preincubation, at timed intervals, 50 μL of 5 mM p‐nitrophenyl‐α‐D‐glucopyranoside solution in 0.1 M phosphate buffer (pH 6.9) was added to each well. The reaction mixtures were incubated at 25°C for 5 min. The absorbance was recorded at 405 nm and compared to that of the control. The IC_50_ was expressed as an inhibitory of 50%. The α‐glucosidase inhibitory activity was expressed as inhibition percent and was calculated as follows.

$$\%\text{inhibition}=\frac{{\mathrm{Abs}}_{\text{control}}-{\mathrm{Abs}}_{\text{sample}}}{{\mathrm{Abs}}_{\text{control}}}\times 100\%$$

### 2.4 In vivo Diabetic Inhibitory Activity of the Samples

#### 2.4.1 Experimental Animals

In this experiment, 18 male mice of Swiss albino (*Mus musculus*) weighing between 20 and 25 g were used for this experiment; the animals were bought from the Pasteur Institute of Ho Chi Minh City. The male mice were randomly divided into six groups of three animals each. Before tests, mice will be familiarized with the new environment for 7 days, with 12 h of light and 12 h of darkness. The mice had unlimited access to tap water.

After the mice got used to the living conditions, diabetic mice were created by a single intraperitoneal injection of alloxan 135 mg/kg body weight in normal saline (prepared immediately before the experiment). Forty‐eight hours later, blood samples were obtained by pricking the lateral tail vein using a sterile needle to determine the fasting blood glucose index (IG) via a glucometer (ACCU‐CHEK Active, Germany) and confirm diabetes. The IG value of mice around 300–400 mg/dL was accepted for diabetes and used for the experiments (Ajiboye, Shonibare, and Oyinloye 2020). Alloxan‐induced diabetic groups were cured with gliclazide (10 mg/kgBW) (Abdelkader et al. 2020) and samples with three concentrations: 300, 400, and 500 mg/kgBW (Begashaw, Dagne, and Yibeltal 2023; Kumar et al. 2021). Based on the particular individual body weight of mice, we calculated the amount of gliclazide and investigated the samples of corm, fruit, seed, and inflorescence to gain the required concentrations of 10, 300, 400, or 500 mg/kgBW in doses, respectively.

Diabetic mice drank 0.1 mL samples twice a day for 20 days of treatment. This study was approved by the Ho Chi Minh City University of Science—HCMUS with approval number 603/KHTN‐ACUCUS. The animals were grouped as shown in Table 1. The samples were administered orally to diabetic mice, and the experiment lasted for 20 days.

**TABLE 1:** Experimental grouping of animals.

No.	Groups	Animals
1	Group 1 (G1)	Normal control mice
2	Group 2 (G2)	Untreated diabetic mice
3	Group 3 (G3)	Diabetic mice treated with gliclazide (10 mg/kgBW)
4	Group 4 (G4)	Diabetic mice treated with samples (300 mg/kgBW)
5	Group 5 (G5)	Diabetic mice treated with samples (400 mg/kgBW)
6	Group 6 (G6)	Diabetic mice treated with samples (500 mg/kgBW)

For the hypoglycemia test, mice were immobilized in a special tube, and blood was drawn from the tail vein after sterilization. During the test, the blood glucose levels were recorded on days 1, 5, 9, 15, and 20 by the glucometer. At the end of the experiments, the animals were killed, and the pancreas was dissected and immediately processed in 10% formalin solution using paraffin techniques. Sections with a thickness of 5 μm were sliced and dyed with hematoxylin. Phlox was examined histologically. A light microscope was used to make histological observations. The mice underwent testing and then were taken to the veterinary facility for euthanasia and dismantled as medical waste after the experiments were finished.

### 2.5 Statistical Analysis

The mean and standard deviation were calculated using descriptive statistics, and their significant differences (*p* < 0.05) were evaluated using analysis of variance (ANOVA) and Tukey's test for multiple comparisons. The variance was analyzed using IBM SPSS Statistics 20 software. Statistical comparisons between normal and the treatment groups were performed by one‐way analysis of variance (ANOVA). The values are presented as mean ± SD.

## 3 Results and Discussion

### 3.1 In vitro Hyperglycemic Inhibition of *M. balbisiana* Parts

**FIGURE 1:**
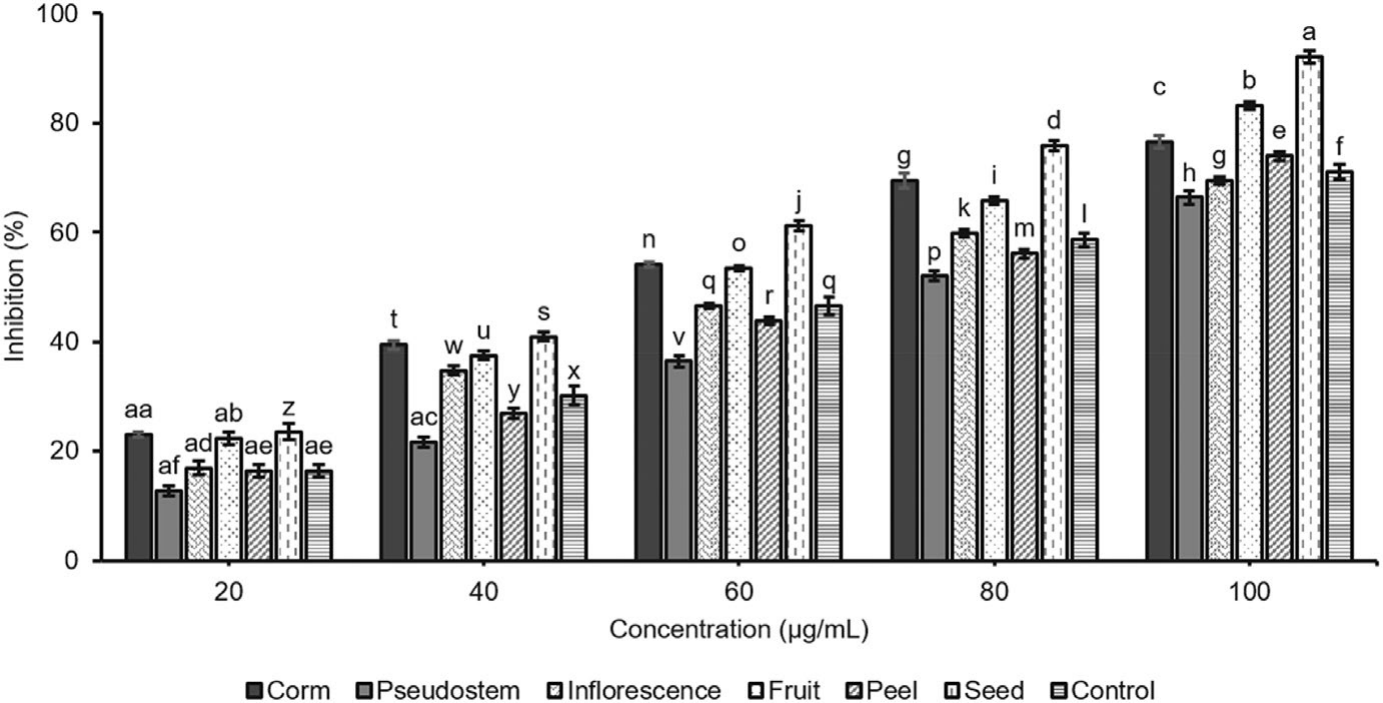
α‐amylase inhibition by extracts of different parts of *M. balbisiana*. In each bar, different letters in the bars represent a statistically significant difference at *p* < 0.05 according to ANOVA analysis.

**FIGURE 2:**
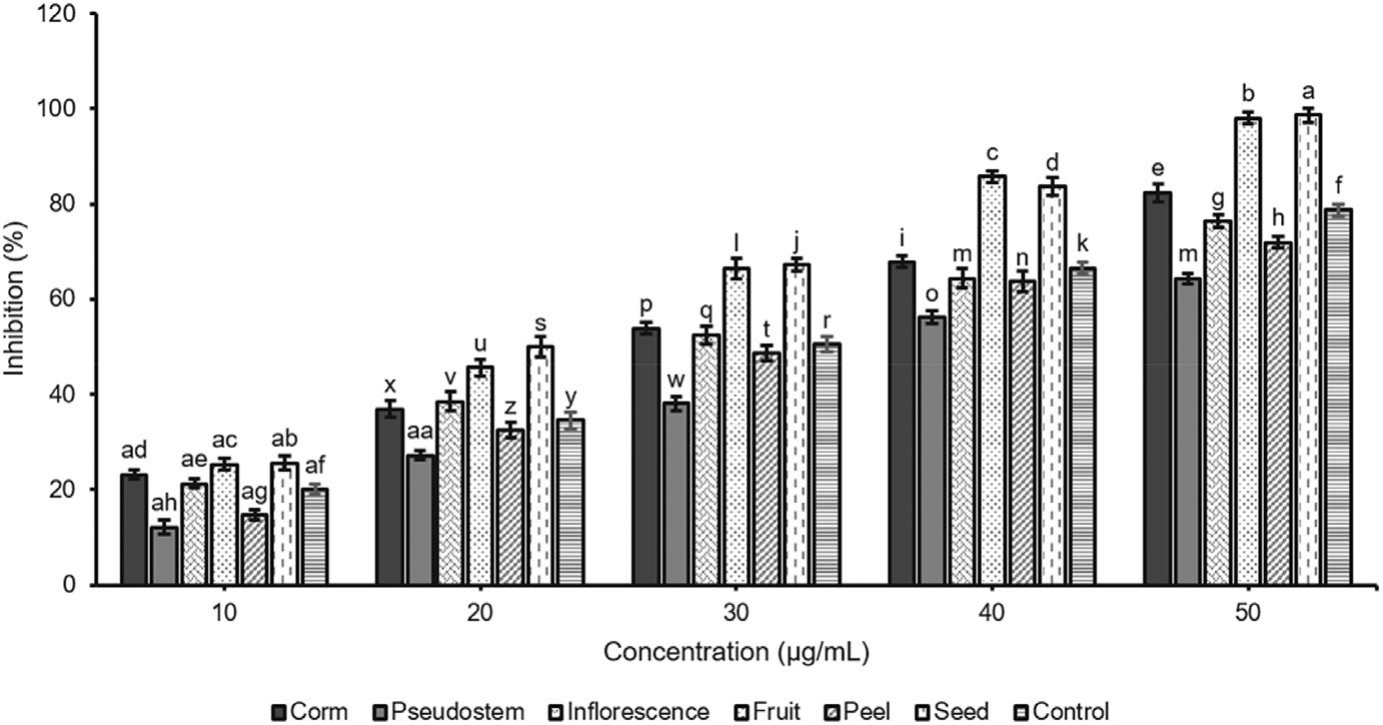
α‐glucosidase inhibition by extracts of different parts of *M. balbisiana*. In each bar, different letters in the bars represent a statistically significant difference at *p* < 0.05 according to ANOVA analysis.

α‐amylase and α‐glucosidase digestive enzymes are mostly important for the breakdown of carbohydrates into monosaccharides to enable their easy absorption. α‐amylase plays a vital role in breaking down polysaccharides into oligosaccharides. Subsequent digestion is carried out by the enzyme α‐glucosidase, which hydrolyzes oligosaccharides to monosaccharides, such as fructose and galactose, alongside glucose. The small intestine would absorb it into the hepatic portal vein (Bushnak, El Hajj, and Jaber 2021). In type 2 diabetes, the IG value increases, which is the first sign of a metabolic abnormality. Inhibiting these enzymes is a key therapeutic goal for managing hyperglycemia. The consumption potential α‐glucosidase and α‐amylase inhibitors can effectively control the postprandial blood sugar level. In this study, the inhibitory activity of α‐amylase and α‐glucosidase enzymes of the samples of the corm, pseudostem, inflorescence, fruit, peel, and seed from *M*. *balbisiana* was investigated (Figures 1 and 2).

The samples of the corm, pseudostem, inflorescence, fruit, peel, and seed from *M*. *balbisiana* had a concentration‐dependent manner on α‐amylase inhibition with IC_50_ values of these surveyed samples were 58.16 μg/mL (corm), 76.89 μg/mL (pseudostem), 67.31 μg/mL (inflorescence), 57.86 μg/mL (fruit), 69.07 μg/mL (peel), 51.29 μg/mL (seed), 68.06 μg/mL (acarbose—control) (Figure 1). The seed sample showed the highest inhibition, which followed by fruit, corm, inflorescence, peel, and pseudostem, respectively.

Figure 2 shows the in vitro α‐glucosidase inhibitory activity of six selected parts of *M*. *balbisiana*. The IC_50_ values of the corm, pseudostem, inflorescence, fruit, peel, seed, and the control were 28.64, 37.73, 30.05, 23.26, 32.49, 21.63, and 29.94 μg/mL, respectively. Similar to the ability of α‐amylase inhibition, the seed possessed inhibition potential, which was significantly better than that of the standard (Acarbose) and other parts. This inhibition activity had corresponded with the TPC and TSC in these samples. These results also revealed that all selected parts of *M*. *balbisiana* exhibited a remarkable hyperglycemic inhibition, suggesting the presence of potential bioactive components in them. They could inhibit α‐amylase better than that of α‐glucosidase. Some previous studies also indicated that plant‐derived phenolic phytochemicals exhibit α‐amylase inhibitory activity that seems to be stronger than α‐glucosidase (Apostolidis et al. 2006). Besides, seeds tended to pose the highest hypoglycemic effect among studied parts and higher than that of the controls of acarbose. This is extremely important for potential applications in pharmaceutical products or functional foods associated with diabetes treatment. The plant‐based extracts are attractive in medical treatment due to fewer side effects than other synthetic drugs, which is worrying about unwanted causes such as abdominal distention, flatulence, meteorism, and possibly diarrhea. Consequently, the excessive inhibition of pancreatic α‐amylase could bring negative impacts like abnormal bacterial fermentation of undigested carbohydrates in the colon (Apostolidis and Lee 2010; Horii et al. 1986).

Diabetes is caused by a long‐term hyperglycemic state, which is related to lots of microvascular and cardiovascular complications. In diabetic patients, the plasma glucose levels are due to impaired insulin, glucagon secretion, and glucose uptake in the liver and peripheral tissues, as well as the hepatic glucose production. Inhibiting α‐glucosidases is a primary way to reduce antihyperglycemia because this enzyme catalyzes the final step of carbohydrate metabolism. Hyperglycemic inhibitors can exhibit α‐glucosidase activity that prolongs carbohydrate absorption duration and lowers plasma glucose levels. The phytochemical extracts tend to bind to a site other than the active site of the enzyme, which retards the enzyme‐substrate reaction without competing with the substrate at the active site of the enzyme (Ramu et al. 2017).

According to Ramu et al. (2015), banana pseudostem nutraceutical‐rich extract effectively inhibits carbohydrate hydrolyzing enzymes, and its mode of inhibition is mixed type and binds (strong affinity). It contains many essential phytosterols such as β‐Sitosterol, Stigmasterol, and Campesterol. β‐Sitosterol and other phytosterols like Stigmasterol pose potent antidiabetic activity and stimulate insulin secretion, thereby increasing the circulating insulin level for better blood sugar control. Moreover, the in vitro antihyperglycemic activity of *M. balbisiana* parts was poorer than ethanol extract of banana pseudostem from *Musa* sp. Var. Nanjangud rasa bale (IC_50_: 8.11 ± 0.10 μg/mL) (Ramu et al. 2015). Besides, these results agreed with the report of Sharma et al. (2023) that the seed of *M. balbisiana* indicated the highest inhibition against α‐amylase and α‐glucosidase enzymes. However, the inhibition of the investigated digested enzymes of samples of *M. balbisiana* parts in the current study was better than that of their extracts, and the fruit sample revealed higher inhibition compared to its extract in our previous study (Nhon Hoang et al. 2023). This might be because the samples in this study were prepared by fractioning from the extract, which helped to enhance the purity of the phytochemical compounds in the samples.

In short, all parts of *M. balbisiana* showed hyperglycemia effectiveness in vitro studies. Corm, fruit, seed, and inflorescence inhibited α‐amylase and α‐glucosidase enzymes better than two other parts of pseudostem and peel. Hence, it is necessary to continue to evaluate the effects of these parts on in vivo studies with diabetic‐induced mice.

### 3.2 In vivo Antidiabetic Ability of *M. balbisiana* Parts

Among six investigated *M. balbisiana* parts, four of them, including corm, fruit, seed and inflorescence, showed higher ability in in vitro experiments and would be chosen for continuing tests on animal models with six groups of mice arranged in the experimental design in Section 2.4. During the experimental period, the body weight and the fasting blood glucose of mice were monitored. The effects of *M. balbisiana* parts on the body weight and fasting blood glucose of alloxan‐induced diabetic mice are shown in Tables 2 and 3, respectively.

**TABLE 2:** Effects of *M. balbisiana* parts on the body weight of alloxan‐induced diabetic mice.

Samples	Day	G1	G2	G3	G4	G5	G6
Corm	Day 1	22.74 ± 0.42^b^	22.67 ± 0.21^b^	22.56 ± 0.31^d^	22.67 ± 0.17^b^	22.57 ± 0.52^d^	22.91 ± 0.28^c^
	Day 20	26.46 ± 0.52^a^	—	25.53 ± 0.53^b^	24.92 ± 0.73^a^	24.98 ± 0.14^b^	25.51 ± 0.52^ab^
Fruit	Day 1	22.95 ± 0.19^b^	23.31 ± 0.35^a^	22.94 ± 0.27^cd^	22.86 ± 0.22^b^	22.58 ± 0.49^d^	22.45 ± 0.26^c^
	Day 20	26.61 ± 0.49^a^	—	25.87 ± 0.14^b^	25.04 ± 0.44^a^	25.10 ± 0.20^ab^	25.24 ± 0.46^b^
Seed	Day 1	22.78 ± 0.34^b^	22.32 ± 0.31^b^	22.90 ± 0.31^cd^	22.61 ± 0.20^b^	22.76 ± 0.14^cd^	22.87 ± 0.26^c^
	Day 20	26.45 ± 0.27^a^	—	29.25 ± 0.45^a^	24.89 ± 0.15^a^	25.60 ± 0.27^a^	25.86 ± 0.17^ab^
Inflorescence	Day 1	23.07 ± 0.38^b^	23.78 ± 0.33^a^	23.22 ± 0.42^c^	22.92 ± 0.28^b^	23.21 ± 0.42^c^	22.94 ± 0.17^c^
	Day 20	26.69 ± 0.33^a^	—	26.08 ± 0.30^b^	25.17 ± 0.30^a^	25.64 ± 0.33^a^	26.01 ± 0.65^a^

*Note:* For each column, different letters represent a statistically significant difference at *p* < 0.05; G1 (control), G2 (Diabetic mice without treatment), G3 (Diabetic mice treated with Gliclazide), G4, G5 and G6 (Diabetic mice given 300, 400, 500 mg/kg BW).

**TABLE 3:** Effects of *M. balbis*iana parts on fasting blood glucose of alloxan‐induced diabetic mice.

Day	Day	G1	G2	G3	G4	G5	G6
Corm	Day 1	115.67 ± 2.08^ab^	342.67 ± 5.03^bc^	346.67 ± 4.16^b^	373.67 ± 5.50^b^	397.33 ± 8.50^a^	399.67 ± 5.03^a^
	Day 20	118.33 ± 3.51^ab^	—	112.67 ± 3.05^d^	131.33 ± 4.50^e^	136.67 ± 4.16^c^	121.33 ± 4.16^c^
Fruit	Day 1	120.33 ± 1.52^a^	349.66 ± 7.09^b^	347.33 ± 9.60^b^	348.33 ± 4.93^c^	353.6 ± 8.14^b^	357.66 ± 5.13^b^
	Day 20	118.33 ± 5.85^ab^	—	113.33 ± 2.51^d^	123.33 ± 3.51^f^	117.66 ± 3.2^d^	106.33 ± 5.13^d^
Seed	Day 1	117.66 ± 2.51^ab^	337.33 ± 4.72^c^	344.33 ± 3.05^b^	370.00 ± 5.19^b^	355.33 ± 4.50^b^	360.66 ± 7.93^b^
	Day 20	119.16 ± 5.29^ab^	—	118.33 ± 4.04^cd^	126.33 ± 2.64^ef^	113.66 ± 3.21^d^	104.33 ± 2.64^d^
Inflorescence	Day 1	112.00 ± 7.54^bc^	390.33 ± 8.02^a^	391.67 ± 4.04^a^	395.33 ± 3.51^a^	391.33 ± 4.93^a^	392.67 ± 3.21^a^
	Day 20	106.33 ± 5.29^c^	—	125.67 ± 3.79^c^	140.67 ± 3.78^d^	133.67 ± 3.05^c^	129.33 ± 5.69^c^

*Note:* For each column, different letters represent a statistically significant difference at *p* < 0.05; G1 (control); G2 (Diabetic mice without treatment); G3 (Diabetic mice treated with gliclazide); G4, G5, and G6 (Diabetic mice given 300, 400, 500 mg/kg BW).

The results in Table 2 indicate that there is no statistically significant difference among mice groups on the first day for each sample (corm, fruit, seed, and inflorescence). In the control group (G1), the body weight grew significantly in G1 and gradually in G3–6 during the experimental period. A noticeable decrease was seen in G2, and the tested animals died before the test ended.

Table 3 reveals that the oral intake samples of corm, fruit, seed, and inflorescence of *M. balbisiana* reduced the fasting blood glucose level in experimental diabetic mice came back to the standard level compared to the control group after 9–20 days of treatment. The higher concentration of samples (500 mg/kg BW) showed better effectiveness on alloxan‐induced mice than a lower concentration of samples (400 and 300 mg/kg BW) in all investigated samples. The seed sample performed the highest effectiveness in lowering the elevated blood glucose of diabetic mice, followed by fruit, corm, and inflorescence. Seed samples (300, 400, 500 mg/kgBW), fruit samples (400, 500 mg/kgBW), and corm samples (500 mg/kgBW) helped to reduce blood glucose of alloxan‐induced mice to normal levels after 9 days of treatment. Besides, fruit samples (300 mg/kgBW), corm samples (300, 400 mg/kgBW), and inflorescence samples (500 mg/kgBW) returned by day 15, while inflorescence samples (300, 400 mg/kgBW) by day 20. Regarding the seed sample, the percent of blood glucose reduction was 65.60% (G3), 65.94% (G4), 68.01% (G5), and 71.19% (G6) after 9‐day treatment, respectively. With fruit sample, the blood glucose reduced gradually in groups of diabetic mice treated with gliclazide and fruit samples with a reduction of 66.40% (G3), 66.25% (G5), and 70.27% (G6) while fruit sample 300 mg/kg (G4) helped to reduce the glucose level to normal after 15 days of treatment. In terms of experiments with the corm sample, the blood glucose in G2 remained over 300 mg/dL, and they died gradually from day 9 to day 12. In contrast, the glucose level in G3 and G6 turned to the standard level (under 200 mg/dL) by day 9 with a reduction of 67.50% and 69.64%, while two groups (G4, G5) resulted in lower blood glucose of 64.65% and 65.58% to the standard level after 15‐day treatment. In line with the three above samples, the in vivo experiments with inflorescence samples indicated that mice G2 died by day 11 due to lack of treatment. The groups of mice experienced a decrease in blood glucose from over 390 mg/dL to the safe level by day 20 in alloxan‐induce mice with a reduction of 67.91% (G3), 67.06% (G6), after 15 days compared to day 1.

These results indicated that *M. balbisiana* parts have the potential to be applied to functional products that help treat diabetic disorders. The body weight of the untreated diabetic mice group was reduced significantly. This is due to the derangement of metabolic pathways, which is a common feature of diabetes. The loss of body weight of alloxan‐induced mice could be due to muscle waste and loss of tissue proteins. In addition, this also might result from the degeneration of adipocytes and muscle tissues, which may be due to the catabolism of proteins and fats in the body of diabetic mellitus animals (Kalita, Kotoky, and Devi 2016). The increase in body weight of treated mice with samples of parts of *M. balbisiana* at the end of the surveyed period of time may relate to normalize the hyperglycemia to normoglycemia (Ajiboye, Oloyede, and Salawu 2018). This finding indicates that a diet with samples from parts of *M. balbisiana* may stimulate insulin release and encourage peripheral tissue to utilize glucose. It also suggests that the corm, seed, fruit, and inflorescence of *M. balbisiana* exhibited an effective antidiabetic property in alloxan‐induced diabetic mice. The blood glucose level in the treated groups of mice may be due to the pancreatic tissue protective or insulin‐stimulating effect of the surveyed samples from parts of *M. balbisiana*. These results agreed that bract and flower of *M. paradisiaca* had a significant impact on the glycemic profile of diabetic rats following glucose load as opposed to the untreated diabetic group (Vilhena et al. 2020) or that mice were administered 500 mg/kgBW of crude ethanol extract of unripe fruit *M. paradisiaca* resulted in a significant decrease in blood glucose levels and prevented further loss of body weight to some extent (Sarma and Goswami 2018). These results were also consistent with the study of Ramu et al. (2017); Ramu et al. (2015) about the antihyperglycemic effectiveness of flower and pseudostem on normal and diabetic rats.

**FIGURE 3:**
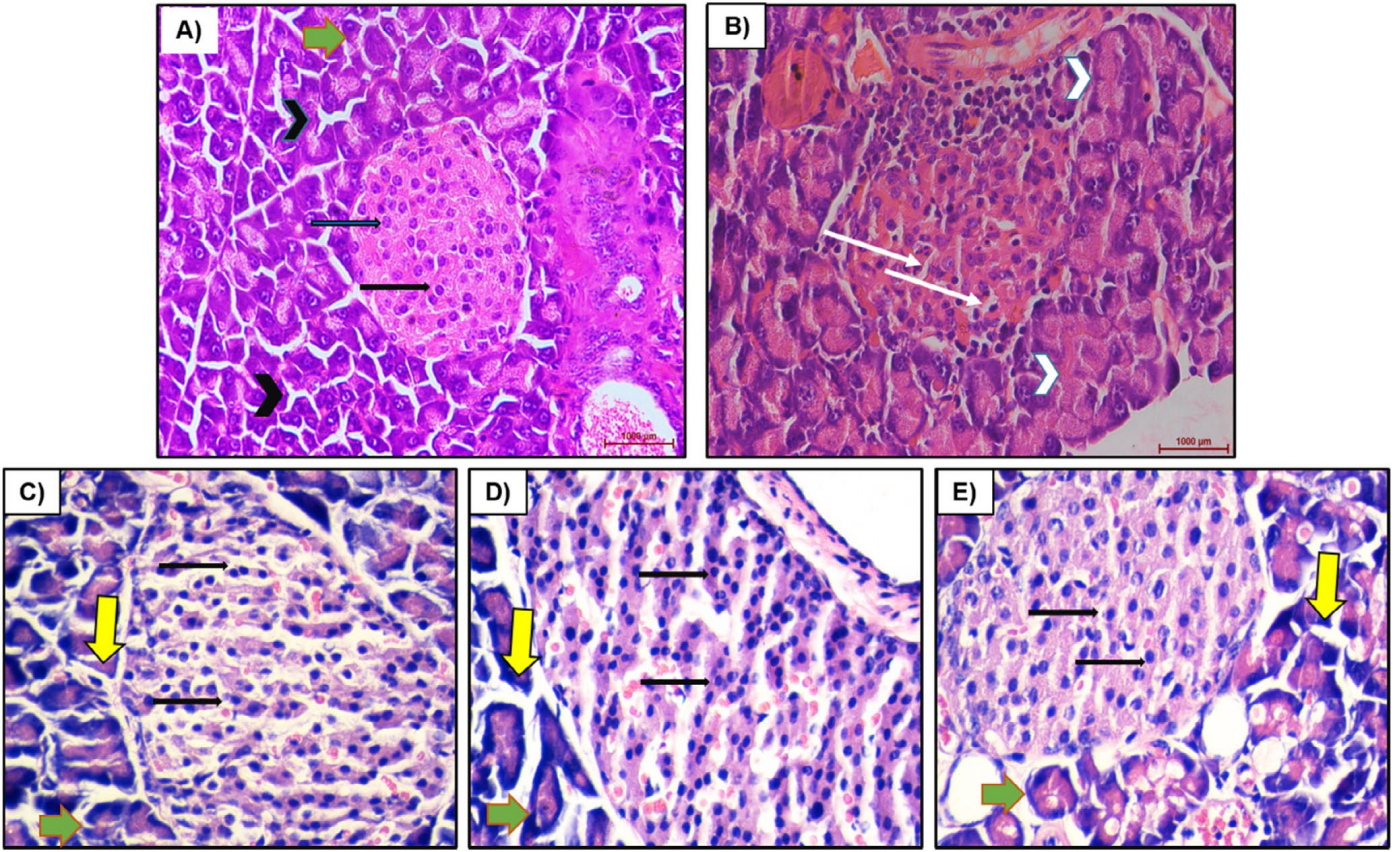
Photomicrograph of a section of the pancreas of normal control at 1000 μm (A), alloxan‐induced mice without treatment at 1000 μm (B), alloxan‐induced mice treated with seed sample 300 mg/kgBW (C), 400 mg/kgBW (D), and 500 mg/kgBW (E).

The pancreatic tissues of the control group, the diabetic mice group without treatment, and the mice group treated with three seed samples (300, 400, and 500 mg/kg) after 20‐day treatment were determined. The results are indicated in Figure 3.

In the normal control mice group, there were no observable microscopic lesions on pancreatic islet cells, congested large blood vessels, and interlobular duct. Photomicrographs of pancreatic sections demonstrating normal pancreatic structure and architecture in the form of normal‐sized islets of Langerhans and normal density of islet cells (black thick arrow) in between normal pyramidal acidophilic pancreatic acini (back arrowheads) (Figure 3A). The pancreas of the untreated diabetic mice group had the infiltration of inflammatory cells into the islets. In contrast, only a few islet cells remained due to complete necrosis, leaving vacuolized areas. In these treatments, the exocrine and endocrine regions could no longer be distinguished due to the deformation of the borders of the islets of Langerhans. Islets were also found less in the pancreatic tissue of the alloxan‐induced mice group than in the control group. The islets of Langerhans experienced depletion and necrobiotic changes in their constituent cells, vacuolated degeneration in most of the cells (white thin arrows), and the exocrine acinar cells exhibit degeneration and dissociation (white arrowheads) (Figure 3B). The photomicrograph of a section of the pancreas of alloxan‐induced diabetic mice administered with 300–500 mg/kgBW of *M. balbisiana* seed sample orally indicates that the areas of pancreatic islet cell were partial restoration and congested blood vessel areas of the pancreas islet cells were restored. There were well‐defined islets of Langerhans with proliferated cell populations (black thick arrow) in between normal pyramidal acidophilic pancreatic acini (yellow thick arrows) and normal exocrine acinar (green thick arrows) (Figure 3C,D,E).

Several available previous reports mention the literature implicating active phytochemicals in plants' response to antidiabetic activities. Plant‐based compounds such as phenols and saponins are considered to be one of the most important plant constituents. There was a linear relationship between total phenol and several bioactivities, and diabetes was included. The hypoglycemic effect of parts of *M. balbisiana* was more pronounced for the corm, seed, and inflorescence at doses of 300, 400, and 500 mg/kg/BW and correlated with the study reported by Ajiboye, Shonibare, and Oyinloye (2020). Bioactive chemical constituents in the plant extract acted as active constituents individually or in a group responsible for the hypoglycemic activity of the plant extracts (Eze et al. 2012). The performance might be because of the high content of polyphenol and saponin compounds in these parts. The higher these active components were also related to the antihyperglycemic activity because they can stimulate insulin release synergistically and/or independently and the repair of pancreatic β cells by the extract or by inhibition of the intestinal absorption glucose (Gad‐Elkareem et al. 2019).

The hyperglycemia phenomenon results from a lack or inadequate incorporation of insulin. Lowering blood glucose level plays an important role in the prevention of microvascular complications (Shanmuga and Subramanian 2012). Alloxan is used in experimental design because it can cause pancreatic injury. It is seen to relate to the DNA molecule of the gene responsible for secreting insulin. Alloxan is harmful to the islet of Langerhans by free radical production since it produces oxygen radicals in the body, while free radicals are one of the mediators in the induction of diabetes mellitus. The production of glucose‐induced protein alterations in patients is associated with oxidative reactions. Antioxidants are generally necessary to protect the cells from reactive oxygen species (Ajiboye, Oloyede, and Salawu 2018).

The results of the current study are consistent with those of many aforementioned research studies. A previous report showed that diabetic rats administered 500 mg/kg and 1000 mg/kg/BW watermelon (*Citrullus lanatus*) juice had effectiveness in an experimental diabetic animal model via observing the decreased insulin and increased glucose levels in STZ‐induced diabetic rats. The oral administration of watermelon juice results in a significant recovery of blood glucose and plasma insulin (Ajiboye, Shonibare, and Oyinloye 2020). In the study of Ara, Tripathy, and Ghosh 2019 *M. balbisiana* flower extract with the dose of 10 mg/100gBW/day effectively enhanced the serum insulin level in STZ‐induced diabetic rats. It exhibited the ability to manage hyperglycemia and oxidative stress in diabetic rats. The remedial effect was observed in diabetic rats treated with an extract from *M. balbisiana* flower that the extract may help to enhance the activities of hexokinase and glucose‐6‐phosphate dehydrogenase in hepatic and skeletal tissue, which results in high expression of Hex‐I gene increased the glucose utilization in a cell. In addition, the extract of *M. balbisiana* flower can help resettle toward the vehicle‐treated control, maybe because it can decrease choleterogenesis. It is considered that *M. balbisiana* flower extract poses an antiapoptotic nature. The activities of SGOT (serum glutamic oxaloacetic transaminase) and SGPT (serum glutamate pyruvate transaminase) recovered in the diabetic group after extract treatment. This reveals that the extract lowers the toxicity present during a diabetic state. In the study of Mallick et al. 2007, aqueous methanolic extract of the root of *M. paradisiaca* at a concentration of 80 mg/100gBW/day to streptozotocin‐induced diabetic rat resulted in a significant remedial effect on IG. In addition, serum insulin level was recovered remarkably after treatment with the extract. This could be because the extract contains active antihyperglycemic agents, which can help to overcome diabetic complications by pancreatic β‐cell regeneration or stimulation of insulin secretion or in other ways. Moreover, after 15 days, rats treated with root extract had a reduction in fasting blood glucose (62.5%), serum total cholesterol (36.2%), triglyceride (54.5%), and low‐density lipoprotein (50.94%) as compared to STZ‐treated animal (Kalita, Kotoky, and Devi 2016). Unripe *M. paradisiaca*‐based diets in alloxan‐induced diabetic mellitus rats reveal that the diet significantly reversed the levels of fasting blood glucose, with a remarkable increase in insulin and glycogen contents. The diet also enhanced the hexokinase activity with a notable reduction in glucose‐6‐phosphatase and fructose‐1‐6‐diphosphatase activities (Ajiboye, Oloyede, and Salawu 2018).

## 4 Conclusion

The findings from this study indicate that parts, namely corm, pseudostem, inflorescence, fruit, peel, and the seed of *M. balbisiana* pose potential hypoglycemic properties, which might play a key role in diabetes management. The obtained results in vitro experiments show that all investigated parts have performed potential hypoglycemic activity on inhibition enzymes of α‐amylase (IC_50_ = 51.29–76.89 μg/mL) and α‐glucosidase (IC_50_ = 21.63–37.73 μg/mL). The results of the in vivo study indicated that samples with different concentrations (300–500 mg/kgBW) could reduce IG in the alloxan‐induced mice model to standard during 9–20 days and in a dose‐dependent manner. The seed sample helped to reduce IG to a normal level after 9‐day treatment, while the time was 9–15 days long for corm and fruit samples and 15–20 days for inflorescence. The obtained results would support the effective further ethnomedical use of this plant to enhance good health, especially in the management of diabetes and its associated complications. These findings provide substantial evidence for the traditional antidiabetic use of *M. balbisiana* parts as well as the probable development of novel antidiabetic medicines.

## Author Contributions

**Thi Ngoc Nhon Hoang:** conceptualization (equal), data curation (equal), formal analysis (equal), investigation (equal), methodology (equal), resources (equal), software (equal), writing – original draft (equal), writing – review and editing (equal). **Quang Liem Nguyen:** data curation (equal), formal analysis (equal), investigation (equal), resources (equal), software (equal). **Thi Thanh Ngan Le:** data curation (equal), investigation (equal), software (equal). **Ngoc Hoa Vo:** data curation (equal), investigation (equal), software (equal). **Thi Anh Dao Dong:** conceptualization (equal), methodology (equal), supervision (equal), validation (equal), writing – original draft (equal), writing – review and editing (equal). **Thi Hong Anh Le:** conceptualization (equal), funding acquisition (equal), methodology (equal), supervision (equal), validation (equal), writing – original draft (equal).

## Conflicts of Interest

All authors have read and agreed to the published version of the manuscript. The authors declare no conflict of interest.

## Data Availability

The data that support the findings of this study are available from the corresponding author upon reasonable request.
