# Correction to “Comparative Study on the Hypoglycemic Effects of Different Parts of *Musa balbisiana*”

**DOI:** 10.1002/fsn3.70070

**Published:** 2025-03-04

Hoang, T. N. N., Nguyen, Q. L., Le, T. T. N., Vo, N. H., Dong, T. A. D., & Le, T. H. A. (2024). Comparative Study on the Hypoglycemic Effects of Different Parts of *Musa balbisiana* . Food Science & Nutrition, 12(12), 10,347–10,356. https://doi.org/10.1002/fsn3.4573.

1. In section 2.2. Samples Preparation:

The following paragraph was missing a sentence: “To determine the diabetic properties of parts of *M. balbisiana* , these parts, including corm, inflorescence, fruit, peel, and seed, were extracted according to the protocol in our previous study (Nhon Hoang et al. 2023). Briefly, powder materials were added with 70% methanol, with the material/solvent ratios 1/20 w/v, and put in a water bath at 60°C for 120 min. After that, the obtained mixtures were centrifuged at 5500 rpm for 15 min and then filtered through the Whatman No. 4 filter paper to have homogenous extracts. Fresh pseudostem was pressed to obtain pseudostem juice. The obtained powder of corm, pseudostem, inflorescence, fruit, peel, and seed was diluted by dimethyl sulfoxide (DMSO) to have samples in different concentrations for the in vitro experiments and will diluted in distill‐water for in vivo experiments.”

This should be revised as:

“To determine the diabetic properties of parts of *M. balbisiana* , these parts, including corm, inflorescence, fruit, peel, and seed, were extracted according to the protocol in our previous study (Nhon Hoang et al. 2023). Briefly, powder materials were added with 70% methanol, with the material/solvent ratios 1/20 w/v, and put in a water bath at 60°C for 120 min. After that, the obtained mixtures were centrifuged at 5500 rpm for 15 min and then filtered through the Whatman No. 4 filter paper to have homogenous extracts. Fresh pseudostem was pressed to obtain pseudostem juice. **Next, the gained extracts and juice were partitioned with a chloroform‐methanol solvent system to remove lipids and other pigments and select the fractions with high content of total polyphenols, flavonoids, and saponins for freeze‐drying to obtain samples**. The obtained powder of corm, pseudostem, inflorescence, fruit, peel, and seed was diluted by dimethyl sulfoxide (DMSO) to have samples in different concentrations for the in vitro experiments and will diluted in distilled water for in vivo experiments.”

2. In the Abstract, the sentence “Seed poses the highest capacity among surveyed parts on α‐amylase (IC50: f μg/mL) and α‐glucosidase (IC_50_: 21.63 μg/mL) as well as effectively lowers the blood glucose index (IG) in alloxan‐induced mice” was incorrect. It should be revised to “Seed poses the highest capacity among surveyed parts on α‐amylase (IC50: **51.29** μg/mL) and α‐glucosidase (IC_50_: 21.63 μg/mL) as well as effectively lowers the blood glucose index (IG) in alloxan‐induced mice.”.

We apologize for this error.

